# Comparing the effects of oxazepam and diazepam in actual highway driving and neurocognitive test performance: a validation study

**DOI:** 10.1007/s00213-018-4844-5

**Published:** 2018-03-02

**Authors:** S. Jongen, E. F. P. M. Vuurman, J. G. Ramaekers, A. Vermeeren

**Affiliations:** Neuropsychology and Psychopharmacology section, Faculty of Psychology and Neuroscience, Maastricht University, Maastricht, The Netherlands

**Keywords:** Oxazepam, Diazepam, Neurocognitive tests, SDLP, On-the-road highway driving test, Attention

## Abstract

**Objective:**

Screening of drug-induced performance impairment is needed to provide meaningful information for users and prescribers regarding the impact of drugs on driving. The main objective was to assess the effects of oxazepam 10 mg (OXA10), oxazepam 30 mg (OXA30), and diazepam 10 mg (DIA10) on standard deviation of lateral position (SDLP) in a highway driving test in actual traffic and to determine the ability of eight neurocognitive tests to detect comparable effects.

**Methods:**

Twenty-three healthy volunteers participated in a four-way double-blind, placebo-controlled, crossover study. The highway driving test was conducted between 4 and 5 h after drug intake. A range of neurocognitive tests was conducted before and after driving, 2 and 6 h post-treatment, respectively.

**Results:**

Mean SDLP increased by 1.83, 3.03, and 7.57 cm after OXA10, DIA10, and OXA30, respectively. At 2 h post-treatment, all neurocognitive tests, except the useful field of view, showed performance impairment in all active treatments. Effect sizes (ES) were moderate for OXA10, large ES for DIA10, and largest ES for OXA30. Modest correlations were found between changes in SDLP and performance in the attention network test (ANT), the divided attention test (DAT), and the psychomotor vigilance test (PVT).

**Conclusion:**

OXA10 caused minor, DIA10 moderate, and OXA30 severe driving impairment. No neurocognitive test was both dose dependently sensitive and able to be associated with driving impairment. No neurocognitive test can replace the on-the-road highway driving test.

## Introduction

Psychoactive drugs can have side effects, such as sedation and reduced alertness, which can cause driving impairment and increase crash risk (Dassanayake, Michie, Carter, and Jones, 2011; O’Hanlon, Haak, Blaauw, and Riemersma, 1982; Seppala, Linnoila, and Mattila, 1979). Screening a drug’s potential to impair the ability to operate a motor vehicle is a necessary means to provide meaningful precautions for users and prescribers of medicinal drugs (Food Drug Administration, 2015; Kay and Logan, 2011; O’Hanlon, 1986).

Ideally, drug evaluations should follow a tiered approach starting with neurocognitive tests, followed by driving simulators and finally on-the-road tests, as the latter generally have better validity to assess driving impairment (Berghaus and Friedel, 1999; Alvarez and del Río, 2002; Vermeeren, De Gier, and O’Hanlon, 1993; Walsh, Verstraete, Huestis, and Mørland, 2008). The standardized on-the-road highway driving test used in the Netherlands (O’Hanlon, 1984; Ramaekers, 2003; Vermeeren, 2004; Verster and Roth, 2011) is a sensitive and reliable test to assess drug-induced driving impairment. Standard deviation of lateral position (SDLP), the primary outcome measure, has been shown to be sensitive to the effects of many sedative drugs (Leufkens and Vermeeren, 2014; O’Hanlon and Ramaekers, 1995; Ramaekers, 2003; Roth, Eklov, Drake, and Verster, 2014). In addition, SDLP has high validity to predict crash risk, as alcohol-induced changes in SDLP are highly correlated (*r* = 0.99) with alcohol-induced changes in crash risk (Borkenstein et al., 1964; Owens and Ramaekers, 2009).

Initial screening of a drug’s impairing potential can be conducted with neurocognitive tests as these are generally easy to administer and cost-effective. However, such neurocognitive tests should be validated for drug sensitivity and generalizability to actual driving in order to provide reliable outcome measures (ICADTS, 1999; Vermeeren, De Gier and O’Hanlon, 1993; Walsh, Verstraete, Huestis, and Mørland, 2008). A number of neurocognitive tests have been calibrated by several doses of alcohol reaching blood alcohol concentrations (BAC) of 0.2, 0.5, and 0.8 g/L (Jongen, Vuurman, Ramaekers, and Vermeeren, 2014). Results suggested that of the selected tests, the psychomotor vigilance test (PVT) and the divided attention test (DAT) are most promising as initial screening tests to detect drug-induced impairment. A follow-up study found that the PVT, DAT, and attention network test (ANT) were most sensitive to the effects of one night of sleep deprivation and showed moderate associations with driving impairment as measured with changes in SDLP (Jongen, Perrier, Vuurman, Ramaekers, and Vermeeren, 2015).

However, sensitive neurocognitive tests to the effects of alcohol or sleep deprivation are not necessarily able to detect impairing effects of medicinal drugs. For example, the impairment profiles of alcohol and benzodiazepines have been found to differ (Kleykamp, Griffiths, and Mintzer, 2010; Tiplady, Hiroz, Holmes, and Drummond, 2003). The neurocognitive tests selected for the present study were the psychomotor vigilance test (PVT), critical tracking test (CTT), divided attention test (DAT), attention network test (ANT), digit symbol substitution test (DSST), useful field of view test (UFOV), postural balance test (PBT), and the determination test (DT), because these were previously found to be sensitive to the effects of alcohol or sleep deprivation (Jongen et al. 2014, 2015).

The aim of the present study was to assess the sensitivity of a selection of neurocognitive tests to the effects of two medicinal drugs known to impair driving performance, i.e., the benzodiazepines diazepam and oxazepam (Neutel, 1995; Ray, Fought, and Decker, 1992). These medicinal drugs were selected because diazepam 10 mg has been recommended as verum for experimental studies assessing the effects of medicinal drugs on driving (Berghaus et al. 1999). Oxazepam is currently the most frequently prescribed benzodiazepine in many countries (in recommended doses of 10 to 30 mg per day). Recent epidemiological studies have shown that both diazepam and oxazepam are highly prevalent in impaired drivers (e.g., Bezemer et al. 2014).

Thus, the primary objective of the present study was to validate the sensitivity of a range of neurocognitive tests mentioned above to the effects of oxazepam (10 and 30 mg) and diazepam 10 mg and compare it with driving impairment (i.e., SDLP changes in the highway driving test). Secondary aims were to establish mean performance changes in each neurocognitive test associated with each drug and dose for future reference and to determine correlations between drug-induced performance changes in neurocognitive tests and on-the-road highway driving.

## Methods

### Participants

Twenty-three healthy volunteers (12 males, 11 females) aged between 21 and 50 years were recruited through advertisements in local papers and at the University of Maastricht. Initial screening was based on a medical history questionnaire examined by the medical supervisor. Eligible participants were invited for a physical examination, which included urinalysis, tests for drugs of abuse (amphetamines, benzodiazepines, cannabis, cocaine, 3,4-methylenedioxymethamphetamine, and opiates), and a 12-lead electrocardiogram. The following inclusion criteria had to be met: possession of a valid driving license for 3 years or more, driving experience of at least 5000 km per year on average over the last 3 years, and a body mass index (BMI) between 19 and 29 kg m^−2^. Exclusion criteria included the following: shift work; history of a sleep disorder; extreme morning or evening type as measured with the Morning Evening Questionnaire (MEQ; Horne and Ostberg, 1976); any history of psychiatric or medical illness; history or current drug or alcohol abuse; current use of psycho-active medication; excessive caffeine use, defined as drinking six or more cups of coffee per day.

The mean (± SD) age of the participants was 36.8 (± 9.5) years. The study was conducted in accordance with the code of ethics on human experimentation established by the declaration of Helsinki (1964) and amended in Seoul (2008). All participants were informed about the study’s goal, procedures, and potential hazards in writing, and they gave their informed consent in writing. The Medical Ethics Committees of Maastricht University approved the study. Participants received a financial compensation for their participation in the study.

### Design

The study was conducted according to a 4-way, randomized, double-blind, placebo-controlled, crossover design. The four treatment conditions were single oral doses of oxazepam 10 mg (OXA10), oxazepam 30 mg (OXA30), diazepam 10 mg (DIA10), and placebo (PBO). Order of treatment conditions was balanced over participants by using a William design. Washout periods between treatments were at least 7 days. To reduce order effects of neurocognitive tests, two neurocognitive test sequences were applied and these were balanced over participants.

### Procedure

Participants were individually trained to perform the behavioral tests prior to the first treatment day. Participants agreed not to use any drugs of abuse or oral medication (except oral contraceptives and paracetamol) during the study. During participation in the study, alcohol intake was not allowed from 24 h prior to each test day until discharge. On treatment days, caffeine intake and smoking were not allowed until discharge.

**Fig. 1:**
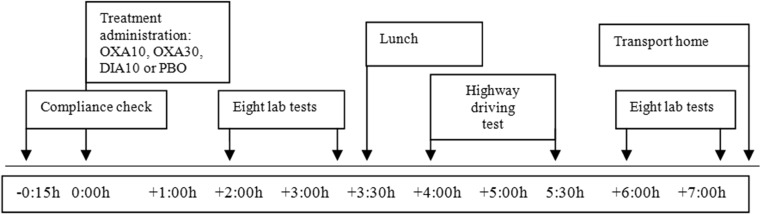
Timeline of a treatment day. Time points are relative to time of treatment administration

On treatment days, participants arrived at the testing site at 8.45 or 10.00 h. The Groningen Sleep Quality Scale (Mulder-Hajonides van der Meulen, 1981) was administered to assess sleep quality, and urine and breathe samples were yielded to assess compliance with use of drugs and alcohol. Four participants were tested on each testing day. Participants ingested a single treatment dose at 9:00 am, 9:05 am, 10:15 am, or 10:20 am. Four hours after drug intake, the standardized highway driving test was conducted. Before and after the driving test, i.e., 2 and 6 h after treatment intake, participants performed two sessions of laboratory testing (session 1 and session 2). Each session consisted of the PVT, CTT, DAT, ANT, DSST, UFOV, PBT, and DT. Before the driving test, a standardized light lunch was served. After completion of session 2 (at 4:00 pm or 5.15 pm), participants were transported home by study personnel. See Fig. 1 for a timeline of the study procedures.

### Assessment

#### Highway driving test

In the standardized highway driving test (O’Hanlon, 1984; Verster and Roth, 2011), the participant operates a specially instrumented vehicle for approximately 1 h over a 100-km (61-mile) primary highway circuit (A2 Netherlands, Maastricht-Weert v.v.), accompanied by a licensed driving instructor having access to dual controls. The task of the participant is to maintain a constant speed of 95 km/h (58 m/h) and a steady lateral position between the delineated boundaries of the right traffic lane. The vehicle’s speed and lateral position are recorded continuously. These signals are digitized at a rate of 4 Hz and edited off-line to remove data recorded during overtaking maneuvers or disturbances caused by roadway or traffic situations. The remaining data are then used to calculate mean values and standard deviation of lateral position and speed. The primary outcome variable is standard deviation of lateral position (SDLP, in cm) which is a measure of road tracking error or ‘weaving’.

#### Psychomotor vigilance test

The psychomotor vigilance test (PVT) is based on a simple reaction time test (Dinges and Powell, 1985). Dependent variables are mean reaction time (RT in ms) and number of lapses (RT > 500 ms). In addition, inverse reaction times (I/RT) were calculated, as it emphasizes slowing in the optimum and intermediate response domain and substantially decreases the contribution of long lapses (Basner and Dinges, 2011). Test duration is 10 min.

#### Critical tracking task

The critical tracking task (CTT) measures the ability to control an unstable error signal using a joystick in a first-order compensatory tracking task (Jex, McDonnell, and Phatak, 1966). The frequency of cursor deviations at which the participant loses control is the critical frequency or lambda (λ_c_ in rad sˉ1). Test duration is approximately 3 min.

#### Divided attention task

The divided attention task (DAT) measures the ability to divide attention between two simultaneously performed tasks, a tracking task and a visual target detection task (Moskowitz, 1973). The primary dependent measures in the respective subtasks are tracking error (in mm) and average reaction time to targets (in ms). Secondary control measures are number of control losses in the tracking task and number of hits in the target detection task. Task duration is 12 min.

#### Digit symbol substitution test

The digit symbol substitution test (DSST) measures processing speed and working memory. A computerized version (McLeod, Griffiths, Bigelow, and Yingling, 1982) of the original paper-and-pencil test taken from the Wechsler Adults Intelligence Scale was used. The performance measure is the number of digits encoded correctly within 3 min.

#### Attention network test

The attention network test (ANT) is a choice reaction time using different warning cues (no cue, double cue, center cue, spatial cue) and target stimuli (with congruent or incongruent flankers). It provides measures of three functions of attention, i.e., alerting, orienting, and executive attention (Fan, McCandliss, Sommer, Raz, and Posner, 2002). Dependent variables are total reaction time, alerting effect (i.e., RT no cue–RT double cue), orienting effect (RT center cue–RT spatial cue), and conflict effect (RT incongruent flankers–RT congruent flankers). The test duration is approximately 20 min.

#### Postural balance test

The postural balance test (PBT) is measured by using the AMTI AccuSway System (Advanced Mechanical Technology, Inc., Watertown, MA) force platform. Postural sway is assessed by the area of the 95% confidence ellipse enclosing the center of pressure (A95 in cm^2^). The test is conducted with feet apart at hip’s width in two trials of 60 s: one trial with the participants’ eyes open and one trial with eyes closed.

#### Determination test

The determination test (DT) (Schuhfried, 2005) is a choice reaction time task measuring resilience of attention and reaction speed under conditions of sensory stress. The task is to identify various visual and auditory stimuli and to react to them by pressing the respective corresponding response buttons, using the response panel of the Vienna Test System. Median reaction time and correct responses were used to assess performance. Total duration of the test is 4 min.

#### Useful field of view test

The test of useful field of view (UFOV) is a computer-based test measuring detection time for three subtests (visual processing speed, divided attention, and selective attention) which involve attentional tasks of increasing difficulty (Edwards et al., 2005). Total detection time was computed by summing the threshold scores for the three subtests. Total duration of the test is approximately 7 min.

### Statistical analyses

Sample size calculation was based on detecting a minimally relevant difference with an effect size of 0.25 in SDLP, the primary measure of this study. Given a test-retest reliability of SDLP of at least *r* = 0.70, a group of 22 participants should permit detection of a mean change of 2.0 cm, with a power of at least 90% and an *α* of .05. Using a Williams design to achieve balance in four treatment orders, a total of 24 participants were needed.

SDLP in the highway driving test was analyzed using general linear model (GLM) for repeated measures with treatment (OXA10, OXA30, DIA10, PBO) as within-subject factor. Three paired sample *t* tests were conducted to assess the drug-placebo contrasts. Each parameter of the neurocognitive tests was analyzed using a 2 × 4 GLM repeated measures with treatment (OXA10, OXA30, DIA10, PBO) for sessions 1 and 2 separately. If a significant treatment effect was found, three paired sample *t* tests were conducted between each treatment and placebo. If the model assumptions were violated, a suitable transformation was selected for analysis; if the assumption remained violated, a nonparametric method (i.e., Friedman test for a main effect and Wilcoxon signed-rank test for simple effects) was selected.

Change scores for each of the dependent variables were transformed to z-scores, which were calculated across the pooled changes in the active treatment conditions relative to placebo. This allows for easy comparison across each of the various performance tests (Dry, Burns, Nettelbeck, Farquharson, and White, 2012). In addition, to compare the magnitudes of the drug-placebo differences between tests and parameters, effect size (ES) statistics for repeated measure designs were calculated (i.e. t_c_[2(1-r)/n]^1/2^; Dunlap, Cortina, Vaslow, and Burke, 1996). Effect sizes between 0 and 0.19 are considered small, between 0.20 and0.69 are considered moderate, and 0.70 or higher are considered large (Lakens, 2013).

Finally, Pearson’s correlations were used to correlate change scores in session 1 and session 2 for each of the dependent variables with drug-placebo changes in SDLP. All statistical analyses were done by using the Statistical Package for the Social Sciences for Windows (version 21; SPSS Inc., Chicago, IL, USA).

## Results

### Missing data

One male participant did not participate in the OXA10 condition, due to reasons unrelated to the study drug. After DIA10, one female participant discontinued testing 3 h after drug intake, due to nausea. Consequently, no data were collected for this participant for the ANT in session 1, the driving test, and all neurocognitive tests in session 2. Due to technical problems, no data of the balance test were available for one participant in the eyes open condition in session 2 after OXA10. The dataset of one male participant was removed from statistical analysis, because of non-compliance in the PBO condition. Extreme outliers, defined as values below the first or above the third quartile, were removed from the respective parameter. In the ANT, two outliers were identified, one in the orienting and one in the conflict effect. In the UFOV test, eight outliers were identified in seven participants. In the PBT, one outlier was identified in the eyes closed condition.

### Highway driving test

Nineteen of 90 driving tests (21.1%) were prematurely terminated. These driving tests were either terminated by the driving instructor as he judged the participant to be too drowsy to continue safely (i.e., in nine cases; two times after OXA10, two after DIA10, and five after OXA30) of by the participants when they felt to be too drowsy to continue safely (i.e., in ten cases: two times after OXA10, three after DIA10, and five after OXA30). In all of these cases, mean SDLP scores were calculated from the data collected up to the termination of the test.

**Fig. 2:**
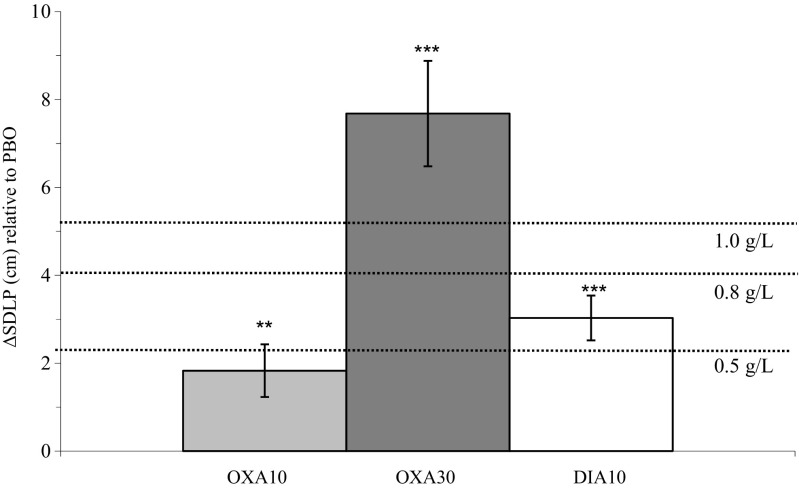
Mean changes of standard deviation of lateral position (ΔSDLP) after oxazepam 10 mg (OXA10), oxazepam 30 mg (OXA30), diazepam 10 mg (DIA10) relative to placebo (PBO). **p < 0.01, ***p < 0.001. *Error bars* indicate the standard error of the mean. To indicate clinical relevance, cut-off values of SDLP changes at a blood alcohol concentration of 0.5, 0.8, and 1.0 g/L are added (Louwerens et al. 1987)

Table 1 presents the mean (SE) of the mean SDLP scores. Analysis of variance showed a significant difference between treatments (F_3,17_ = 19.67, *p* < 0.001). Figure 2 shows the mean changes (i.e., ΔSDLP) from PBO compared to OXA10, DIA10, and OXA30. The mean SDLP score presented in Table 1 include all participants (i.e., *n* = 22, *n* = 21, *n* = 22, *n* = 21, for PBO, OXA10, OXA30, and DIA10, respectively). Because of missing data for one participant in the OXA10 and DIA10 condition, respectively, mean SDLP changes of OXA10 and DIA10 in Table 2 are calculated based on *n* = 21. Figure 2 shows mean SDLP changes of + 1.83, + 3.03, and + 7.57 cm for OXA10, DIA10, and OXA30, respectively, indicating ΔSDLP comparable to a BAC of < 0.5, 0.5–0.8, and > 0.8 g/L, respectively.

**Table 1:** Mean (SE) of laboratory tests, overall effects of treatment, and paired sample *t* tests

		Mean (SE)				Overall effect		Paired *t* tests		
Test	Time of testing	OXA10 (*n* = 21)	OXA30 (*n* = 22)	DIA10 (*n* = 21)	PBO (*n* = 22)	*F*	*P*	OXA10-PBO	OXA30-PBO	DIA 10-PBO
Highway driving test										
SDLP (cm)	+ 4 h	18.47 (0.90)	24.15 (1.36)	19.47 (0.89)	16.58 (0.65)	19.67	< 0.001	< 0.01	< 0.001	< 0.001
Psychomotor vigilance test										
Inverse reaction time	+ 2 h + 6 h	4.07 (0.09) 4.13 (0.09)	3.35 (0.14) 3.85 (0.13)	3.86 (0.10) 4.13 (0.10)	4.14 (0.09) 4.13 (0.11)	13.40 NS	< 0.001	–	< 0.001	< .001
Lapses (#)*	+ 2 h + 6 h	0.90 (1.38) 1.19 (0.72)	11.86 (3.15) 4.77 (1.79)	2.29 (0.74) 0.90 (0.31)	0.64 (0.16) 0.77 (0.29)	χ ^2^ = 38.87 χ ^2^ = 10.29	< 0.001 < 0.05	–	< 0.001 < 0.01	< 0.05-
Mean reaction time (ms)	+ 2 h + 6 h	258 (7) 254 (6)	454 (68) 311 (34)	277 (9) 255 (7)	251 (5) 255 (7)	4.61 NS	< 0.05	–	< 0.01	< 0.01
Critical tracking test										
Critical lambda (rad/s)	+ 2 h + 6 h	3.08 (0.12) 3.01 (0.14)	2.49 (0.13) 3.00 (0.13)	3.00 (0.09) 3.26 (0.11)	3.34 (0.10) 3.23 (0.11)	13.17 NS	< 0.001	< 0.05 -	< 0.001 -	< 0.01 -
Divided attention test^a^										
Primary task: z-AE + z-lg10(CL)	+ 2 h + 6 h	− 0.09 (0.46) − 0.53 (0.38)	1.97 (0.53) 0.32 (0.45)	0.82 (0.36) − 0.18 (0.39)	− 0.69 (0.36) − 0.63 (0.38)	11.15^a^ NS^a^	< 0.01	–	< 0.001	< 0.01
Secondary task: reaction time (ms)	+ 2 h + 6 h	1966 (91) 1892 (81)	2323 (91) 2105 (70)	2152 (61) 1975 (80)	1883 (65) 1859 (74)	16.33^a^ NS^a^	0.001	–	< 0.001	< 0.001
Attention network test										
Mean total reaction time (ms)	+ 2 h + 6 h	584 (21) 558 (21)	730 (28) 626 (26)	630 (26) 594 (24)	547 (18) 560 (22)	26.48 6.67	< 0.001 < 0.01	0.001 -	< .001 < 0.001	< 0.001 < 0.05
Conflict effect (ms)	+ 2 h + 6 h	124 (10) 120 (10)	148 (13) 128 (8)	125 (7) 127 (9)	105 (6) 116 (7)	5.57^b^ NS	< 0.01	< 0.05	< 0.01	< 0.05
Orienting effect (ms)	+ 2 h + 6 h	49 (5) 54 (5)	68 (12) 63 (6)	63 (5) 51 (6)	47 (5) 51 (5)	9.82^b^ NS	0.001			< 0.01
Alerting effect (ms)	+ 2 h + 6 h	53 (7) 48 (6)	71 (8) 64 (8)	58 (9) 61 (7)	63 (6) 52 (6)	NS NS				
Digit symbol substitution test										
Correct responses (#)	+ 2 h + 6 h	92.05 (2.97) 99.33 (2.64)	73.41 (3.05) 89.95 (2.26)	88.95 (2.83) 96.48 (2.89)	97.59 (2.23) 98.09 (2.89	22.71 6.80	< 0.001 < 0.01	< 0.01 -	< 0.001 0.001	< 0.001 -
Useful field of view ^c^										
Total detection time (ms)	+ 2 h + 6 h	89 (7) 86 (6)	161 (23) 95 (6)	105 (10) 92 (9)	107 (14) 85 (7)	4.51^c^ NS^c^	0.02	–	0.02	–
Postural balance test										
Eyes open–ln area 95 (cm^2^)	+ 2 h + 6 h	0.69 (0.09) 0.38 (0.09)	1.48 (0.18) 0.87 (0.14)	0.79 (0.15) 0.90 (0.10)	0.26 (0.09) 0.51 (0.10)	10.10 5.88	< 0.001 < 0.01	< 0.01 -	< 0.001 < 0.05	0.01 0.001
Eyes closed–ln area 95 (cm^2^)	+ 2 h + 6 h	1.01 (0.14) 0.68 (0.10)	1.58 (0.19) 1.25 (0.15)	1.02 (0.17) 0.80 (0.13)	0.70 (0.10) 0.71 (0.09)	6.56^b^ 6.76	< 0.01 < 0.01	- -	< 0.001 < 0.001	–
Determination test										
Median reaction time (ms)	+ 2 h + 6 h	740 (20) 706 (16)	810 (23) 739 (17)	767 (19) 707 (17)	714 (15) 689 (15)	8.14 NS	0.001	< 0.05	< 0.001	< 0.05 -
Correct responses (#)	+ 2 h + 6 h	262 (9) 279 (7)	230 (11) 254 (10)	256 (8) 281 (7)	273 (8) 279 (9)	5.12 NS	0.01	- -	0.001	- -

*SE* standard error, *OXA10* oxazepam 10 mg, *OXA30* oxazepam 30 mg, *DIA10* diazepam 10 mg, *PBO* placebo, *SDLP* standard deviation of lateral position, *z-AE* z-score of average tracking error, *z-log(CL)* z-score of log transformed number of control losses, *ln* natural log

“*”because assumptions for general linear model were violated, a non-parametric Friedman test was conducted with Wilcoxon signed-rank tests to examine drug-placebo contrasts

^a^*n* = 10

^b^*n* = 19

^c^*n* = 14

**Table 2:** Mean difference scores with 95% confidence intervals with performance after normal night of sleep, mean placebo-normalized absolute z-scores, effect sizes (Dunlap’s) of neurocognitive tests of the mean differences of OXA10, OXA30, and DIA10g compared to placebo and correlations between mean changes of neurocognitive tests and changes in SDLP after OXA10, OXA30, and DIA10

Test		OXA10 vs PLA		OXA30 vs PLA		DIA10 vs PLA		OXA10 vs PLA	OXA30 vs PLA	DIA10 vs PLA	OXA10^a^	OXA30^a^	DIA10^a^
	Time of testing	Δ	[95% CI]	Δ	[95% CI]	Δ	[95% CI]	Effect sizes			*r* ΔSDLP		
Highway driving test													
SDLP (cm)	+ 4 h	1.83	[0.59, 3.08]	7.68	[5.28, 10.08]	3.03	[1.96, 4.10]	0.47^+^	1.39^++^	0.77^++^			
Psychomotor vigilance test													
Inverse reaction time	+ 2 h + 6 h	− 0.07 0.01	[− 0.26, 0.24] [− 0.13, 0.15]	− 0.79 − 0.28	[− 1.03, − 0.55] [− 0.47, − 0.09]	− 0.29 − 0.01	[− 0.42, − 0.15] [− 0.13, 0.12]	- -	1.38^++^ -	0.65^+^ -	- -	- − 0.56**	- -
Lapses	+ 2 h + 6 h	0.29 0.43	[− 0.45, 1.02] [− 0.79, 1.65]	11.23 4.00	[4.74, 17.71] [0.23, 7.77]	1.59 0.10	[0.05, 3.13] [− 0.58, 0.77]	- -	0.95^++^ 0.67^+^	0.69^+^ -	- -	- -	- -
Mean reaction time	+ 2 h + 6 h	10 1	[− 2, 21] [− 10.27, 12.39]	204 56	[64, 343] [− 9, 121]	26 0	[11, 42] [− 10, 10]	- -	0.78^++^ 0.39^+^	0.69^+^ 0.00	- -	- -	- -
Critical tracking test													
Critical lambda	+ 2 h + 6 h	− 0.26 − 0.23	[− 0.50, − 0.02] [− 0.50, 0.04]	− 0.85 − 0.23	[− 1.12, − 0.58] [− 0.48, 0.03]	− 0.34 0.03	[− 0.56, − 0.12] [− 0.15, 0.22]	0.50^+^ -	1.57^++^ -	0.77^++^ -	- -	- -	- -
Divided attenion test													
Primary task: z-AE + z-lg10(CL)	+ 2 h + 6 h	0.57 0.02	[− 0.36, 1.50] [− 0.70, 0.73]	2.90 1.10	[1.77, 4.02] [0.22, 1.99]	1.37 0.47	[0.54, 2.19] [− 0.20, 1.13]	- -	1.71^++^ -	0.90^++^ -	- -	- -	- -
Secondary task: reaction time	+ 2 h + 6 h	117 39	[− 123, 356] [− 114, 191]	567 138	[367, 768] [− 188, 464]	331 80	[169, 493] [− 62, 222]	- -	1.66^+^ −^+^	1.16^++^ -	- -	- -	- -
Attention network test													
Mean total reaction time	+ 2 h + 6 h	45 11	[20, 70] [− 8, 30]	183 67	[142, 224] [38, 95]	82 35	[47, 117] [5, 64]	0.49^+^ -	1.51^++^ 0.56^+^	0.75^++^ 0.31^+^	- 0.58**	- -	- 0.55**
Conflict effect	+ 2 h + 6 h	19 5	[3, 35] [− 11, 22]	45 12	[17, 73] [− 3, 27]	21 11	[5, 37] [− 6, 28]	0.44^+^ -	0.93^++^ -	0.69^+^ -	- -	- -	- -
Orienting effect	+ 2 h + 6 h	6 5	[− 8, 20] [− 8, 18]	23 12	[− 5, 51] [− 5, 30]	18 − 1	[6, 31] [− 20, 17]	- -	- -	0.73^++^ -	- -	- -	- -
Alerting effect	+ 2 h + 6 h	− 10 − 1	[− 27, 8] [− 14, 12]	7 12	[− 10, 24] [− 7, 31]	− 6 11	[− 31, 20] [− 6, 29]	- -	- -	- -	- -	- -	- -
Digit symbol substitution test													
Correct responses	+ 2 h + 6 h	− 6.33 − 0.05	[− 10.37, − 2.29] [− 3.31, 3.21]	− 24.18 − 8.14	[− 29.81, − 18.56] [− 12.52, − 3.76]	− 8.64 − 1.67	[− 12.84, − 4.43] [− 6.20, 2.86]	0.50^+^ -	1.89^++^ 0.65^+^	0.70^++^ -	- -	- -	- -
Useful field of view													
Total detection time	+ 2 h + 6 h	− 17 1	[− 40, 7] [− 10, 12]	59 9	[10, 109] [− 5, 23]	2 4	[− 25, 29] [− 16, 24]	- -	0.69^+^ -	- -	- -	- -	- -
Postural balance test													
Eyes open–ln area 95 (cm^2^)	+ 2 h + 6 h	0.40 − 0.14	[0.14, 0.67] [− 0.39, 0.11]	1.19 0.38	[0.77, 1.61] [0.07, 0.70]	0.47 0.31	[0.10, 0.83] [0.09, 0.54]	1.06^++^	1.90^++^ 0.66^+^	0.95^++^ 0.79^++^	0.60** -	- -	- -
Eyes closed–ln area 95 (cm^2^)	+ 2 h + 6 h	0.28 − 0.07	[− 0.04, 0.61] [− 0.25, 0.12]	0.87 0.56]	[0.45, 1.28] [0.31, 0.82]	0.29 0.03	[− 0.11, 0.69] [− 0.21, 0.27]	+ -	1.26^++^ 0.93^++^	- -	- -	- -	- -
Determination test													
Median reaction time	+ 2 h + 6 h	30 18	[5, 55] [− 1, 37]	97 46	[58, 136] [18, 74]	54 14	[11,96] [− 10, 38]	0.34^+^ -	1.02^++^ -	0.67^+^ -	- -	- -	- -
Correct responses	+ 2 h + 6 h	− 11 − 2	[− 31, 9] [− 16, 13]	− 43 − 25	[− 66, − 20] [− 45, 4]	− 17 − 3	[− 36, 2] [− 14, 8]	- -	0.97^++^ -	- -	- -	- -	- -

*CI* confidence interval, *r* correlation, *SDLP* standard deviation of lateral position, *OXA10* oxazepam 10 mg, *OXA30* oxazepam 30 mg, *DIA10* diazepam 10 mg, *z-AE* z-score of average tracking error, *z-log(CL)* z-score of log transformed number of control losses, *ln* natural log

^a^*n* = 20

^+^moderate effect size, ^++^large effect size. **p < 0.01

### Neurocognitive tests

Table 1 presents a summary of the means and standard errors of the means (SE) of all performance scores and the results of the statistical analyses. In the PBT, A95 scores were not normally distributed and therefore log transformed (e.g., Boyle et al., 2009). After the log transformation, A95 scores were normally distributed. In the PVT, lapses were not normally distributed and therefore analyzed using non-parametric tests (i.e., Friedman test and Wilcoxon signed rank tests).

The DAT was terminated 23 times (12.7%), as participants were unable to complete the test because of motor problems (> 50 control losses): four (OXA10), ten (OXA30), four (DIA10) times in session 1; three times (OXA30) and once (DIA10) in session 2. In addition, the test was once terminated in session 1 after PBO. Secondary control measures were first analyzed. A Friedman test was conducted, as control losses were not normally distributed. A Friedman test showed a statistically significant difference in control losses depending on treatment, χ^2^ (7) = 25.03, *p* = 0.001. A combined score of control losses and tracking error was used to assess performance in the DAT. The distribution of control losses was highly skewed and transformations were applied to its logarithmic scores (log 10) before transformation to z-scores. Log 10 was applied to deal with zero values by using the formula NEWX = LG10 (X + 1.

Repeated measures analyses of variance showed a main effect of treatment in every parameter of the neurocognitive tests, except for orienting and alerting in the ANT. In session 1, all parameters showed impairment after OXA30 and DIA10 compared to PBO, except for the UFOV after DIA10. In session 1 after OXA10, significant impairment was found in all tests, except the PVT, DAT, and UFOV.

In session 2 after OXA30, performance was still impaired in all tests except the CTT and UFOV; after DIA10, only the ANT and PBT with eyes open showed impairment. In session 2 after OXA10, no significant effect in any test was found.

### Effect sizes and correlations

Table 2 shows a summary of mean treatment differences relative to PBO with 95% confidence intervals, Dunlap’s effect sizes, and significant correlations between changes of performance in the neurocognitive tests and ΔSDLP in the highway driving test.

**Fig. 3:**
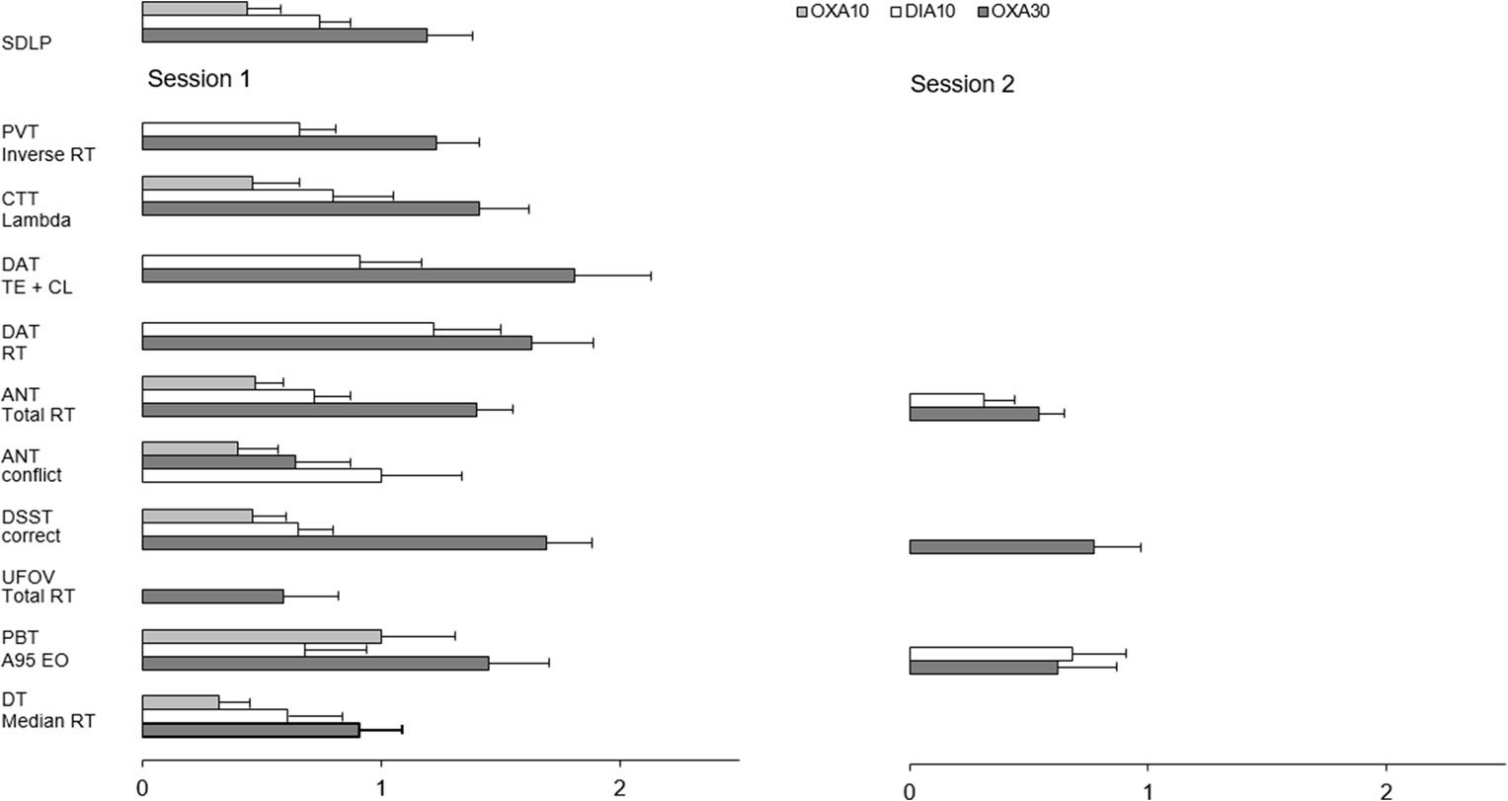
Mean baseline-normalized significant z-scores of oxazepam 10 mg (OXA10), diazepam 10 mg (DIA10), and oxazepam 30 mg (OXA30) compared with placebo across dependent variables of neurocognitive tests. SDLP in the highway driving tests is included as reference test. PVT = psychomotor vigilance test, CTT = critical tracking test, DAT = divided attention test, ANT = attention network test, DSST = digit symbol substitution test, UFOV = useful field of view, PBT = postural balance test, DT = determination test, RT = reaction time, TE = tracking error, CL = control losses, A95 EO = area 95% eyes open. *Error bars* indicate the standard error of the mean

The ES of ΔSDLP in the highway driving test were moderate after OXA10 (0.47) to large after DIA10 and OXA30 (0.77 and 1.39, respectively). In the neurocognitive tests, ES were generally moderate after OXA10, large after DIA10, and largest after OXA30. In addition, ES were larger in session 1 compared to session 2. Figure 3 shows transformed z-scores of change scores for each of the significant dependent variables across the pooled changes in the active treatment conditions relative to placebo. For the PVT and the DT, only the parameters with highest transformed z-scores were reported.

Table 2 depicts correlations between neurocognitive test performance changes and ΔSDLP. These correlations show that significance (i.e., *p* < 0.01) was only found between performance changes in PVT after OXA30 in session 2, in ANT after OXA10 and DIA10 in session 2, and PBT after OXA10 in session 1.

## Discussion

The main aim of the current study was to validate the sensitivity of a range of neurocognitive tests to the effects of OXA10, OXA30, and DIA10 and to compare these effects with driving impairment (i.e., changes in SDLP) during the highway driving test. An increase of SDLP in the highway driving test was found after all three benzodiazepine treatments compared to PBO. After OXA10, a significant SDLP increase of 1.83 cm was found, corresponding to minor impairment as found at a blood alcohol concentration (BAC) of < 0.5 g/L; after DIA10, SDLP increased with 3.03 cm, corresponding to moderate impairment as found at a BAC between 0.5–0.8 g/L; and after OXA30, SDLP increased with 7.57 cm, corresponding to severe impairment as found at a BAC of > 1.0 g/L (Louwerens et al. 1987). In general, neurocognitive test performance 2 h after intake showed comparable impairment as found on SDLP in the highway driving test, while the drug effects were diminished 6 h after intake. At that time, impairment after OXA30 was still significant, but was nearly absent after intake of OXA10 and DIA10.

Based on the mean changes and the magnitude of the effect sizes, several tests (i.e., the ANT, DSST, PBT, and DT) showed to be sensitive to detect mild drug effects after OXA10, which was comparable to changes in SDLP in the highway driving test. These tests are potential candidates to be included in early phase clinical trials to test mild drug effects. As initial tools, neurocognitive tests showing higher levels of impairment are preferable above neurocognitive tests showing lower levels of drug-induced impairment, as generally these tests indicating lower levels of impairment might underestimate the mean drug effect. However, it should be noted that the DSST and ANT were only sensitive to effects of alcohol while BAC was 0.8 g/L, but not at lower doses (Jongen et al., 2014). This indicates that the ANT and DSST seem to be more sensitive to the effect of benzodiazepines as compared to alcohol.

Surprisingly, the DAT and PVT were unable to detect impairment induced by OXA10 and showed lower magnitude of impairment compared to SDLP in the highway driving test. This was rather unexpected as previous studies showed that these tests were sensitive to impairment of low and moderate doses of alcohol (Jongen et al. 2014) and sleep deprivation (Jongen et al., 2015). In addition, the DAT has previously shown to be sensitive to the effects of various medicinal drugs, such as of anxiolytics (Leufkens, Vermeeren, Smink, van Ruitenbeek, and Ramaekers, 2007), hypnotics (Leufkens, Ramaekers, de Weerd, Riedel, and Vermeeren, 2014; Leufkens, Lund, and Vermeeren, 2009; Vermeeren et al., 2002), antihistamines (Vuurman et al., 2004), and antidepressants (Robbe and O’Hanlon, 1995). The failure of the DAT to show significant impairment after OXA10 in the present study might be explained by the large number of missing data, due to termination of four tests. As a result, the dataset might have been underpowered.

In contrast to the DAT, only one previous study used the PVT to assess drug effect on performance (Leufkens et al. 2014). Leufkens et al. (2014) used the PVT and the on-the-road highway driving test to compare the residual effects of zopiclone 7.5 mg and placebo. Results showed that performance in the on-the-road highway driving test was significantly impaired after zopiclone 7.5 mg, but performance in the PVT did not differ from placebo. Together with the results of the present study, this suggests that the PVT and DAT can be a useful tool for initial screening of a moderate to severe drug effect in early phase clinical trials, as these tests are able to detect moderate to large drug effect on sustained and divided attention. However, the PVT lacks sensitivity to mild drug effects, indicating that the use of the PVT in clinical trials might lead to failing to detect mild impairing drug effects on sustained attention. Later phase clinical studies assessing mean drug effects on driving performance should therefore include sensitive measures of on-the-road driving performance (e.g., SDLP) to provide the final evidence of the impairing potential of a drug on driving ability.

The levels of drug-induced impairment found in these neurocognitive tests after intake of these prototypical sedative drugs can be used as thresholds of drug-induced impairment in future studies. A recent review indicated the lack of information regarding clinical relevance of neurocognitive impairment in medicated patient populations (van der Sluiszen et al., 2017). The present results can help to identify those patient groups who are at risk in traffic by, for example, comparing the benchmarks in neurocognitive tests after acute drug administration with long-term use of drugs in patient populations.

The present study found that only 4 out of 96 correlations between driving impairment and neurocognitive test performance were statistically significant. These correlations were a best moderately strong, and found in 3 out of 8 neurocognitive tests, i.e., the PVT, ANT, and PBT. No parameter consistently correlated (i.e., for all three treatment conditions) with driving impairment. In addition, no significant correlations were found between performance changes in the DAT, CTT, DSST, DT, and UFOV, showing that these tests are not associated with driving impairment as measured with changes in SDLP. The results of the present study are in line with previous reviews concluding that only some neurocogntive correlate moderately at best with actual driving impairment (Jongen et al. 2015; Ramaekers, 2003; Verster and Roth, 2012). Overall, neurocognitive tests clearly measure different aspects of drug-induced impairment as compared to SDLP. However, neurocognitive tests may have added value in assessing these drug effects in early phase clinical trials as these are easy to administer and cost-effective.

A limitation of the present study may be that neurocognitive tasks are compared with SDLP to assess drug-induced driving impairment. Although SDLP reflects road tracking control as a fundamental and realistic aspect of driving, it only measures highly automated performance at an operational level as part of driving performance (Michon, 1989). Driving performance also includes risk assessment, decision making and interaction with other road users. The predictive validity of the selected neurocognitive tests to measure drug-induced driving impairment could be underestimated as these tests might measure different relevant aspects of driving performance. Nevertheless, SDLP remains the most valid and sensitive measure to assess a drug’s impairing potential on driving performance.

In conclusion, it was shown that single doses of OXA10 caused minor, DIA10 moderate, and OXA30 severe driving impairment. The DSST, ANT, PBT, and DT were able to detect mild effects of OXA10, but—except for the ANT—were not associated with driving impairment. These tests are potential candidate tests to measure mild drug effects in early phase clinical trials. However, no neurocognitive test is able to replace the on-the-road highway driving test.
